# Impact of SMAASH-C, a novel nutritional supplement, on drug-seeking and toxicity in female and male rats

**DOI:** 10.1038/s41398-024-02940-w

**Published:** 2024-05-27

**Authors:** Eleanor Blair Towers, Ivy L. Williams, Ariadne S. K. Aristidou, Ajibike O. Salako-Akande, Wendy J. Lynch

**Affiliations:** 1 Psychiatry and Neurobehavioral Sciences, University of Virginia, Charlottesville, VA USA; 2 Medical Scientist Training Program, University of Virginia, Charlottesville, VA USA; 3 Getwele Natureceuticals LLC, Halethorpe, MD USA

**Keywords:** Addiction, Neuroscience

## Abstract

Relapse to drug use after abstinence is a major challenge in treating substance use disorder. Exposure to drug-associated cues during abstinence can trigger intense craving and precipitate relapse. New and more effective anti-relapse interventions are critically needed, particularly for cocaine use disorder since no effective pharmacological intervention is available. We discovered that a nutritional supplement we developed as part of a nutritional approach for managing patients with substance use disorder reduced patient reports of drug craving and relapse. The goal of this study was to determine the efficacy of this supplement, SMAASH-C, at reducing drug-craving/relapse vulnerability in males and females in rat models with cocaine. Effects were determined following extended-access cocaine self-administration (24-hr/day for 10 days) and a two-week treatment regimen at a moderate and moderate-to-high dose (0.4 and 0.8 g/kg/day) as well as a 6-week regimen at a moderate dose (0.4 g/kg/day; Experiment 2). We also determined its efficacy to offset serum markers of organ toxicity in response to chronic cocaine self-administration and abstinence (aspartate transaminase, alanine transaminase, amylase; urea nitrogen). In females, both the 2- and 6-week SMAASH-C treatment regimens reduced cocaine-seeking (extinction or cue-induced reinstatement), particularly when drug-seeking was heightened (e.g., during estrus). Despite a lack of efficacy to reduce drug-seeking in males, SMAASH-C treatment normalized cocaine/abstinence-induced increases in serum levels of aspartate transaminase and amylase, which are markers of liver and pancreatic toxicity respectively. Thus, the beneficial effects of oral SMAASH-C treatment over abstinence following chronic cocaine self-administration appears to be sex-specific.

## Introduction

Cocaine use disorder (CUD) is a serious public health concern with approximately 5.1 million regular cocaine users over the age of 12, and 1.3 million individuals with CUD in the United States in 2020 [1]. Despite decades of study and the allocation of a significant amount of resources, no medication has been approved for the treatment of CUD. The lack of medications for CUD is largely due to candidate medications failing in clinical trials despite promising preclinical results. The challenge of failed translation is not unique to CUD research [2]; however, we argue that in order to produce a more representative and translationally relevant body of knowledge on the neurobiological basis and potential treatments for addiction, studies need to use animal models validated to induce addiction-like features like those observed in women and men with CUD and consider biological sex as a variable [3].

Extended-access (extended-access) drug self-administration procedures (i.e., ≥6-hr/day access to the drug) are the gold-standard for inducing addiction-like features in animals [3, 4]. We use an intermittent, extended-access procedure (4 trials/hr; 24-hr/day for 10 days) that was developed to approximate the use patterns observed in humans (e.g., binge/abstinent patterns;[5, 6]. We have confirmed that this procedure induces binge/abstinent patterns of drug intake in both males and females [4], as well as other key features of addiction, including an enhanced vulnerability to relapse assessed using an extinction/cue-induced reinstatement procedure [7], and reversal of this phenotype using treatments known to decrease drug-craving and prevent relapse in humans (e.g., exercise and alternative rewards for cocaine and buprenorphine for fentanyl; [7–10].

Here, using this validated rat model of relapse, we determined the efficacy of oral treatment during abstinence with a novel nutritional supplement, SMAASH-C, at reducing cocaine-seeking and markers of toxicity following extended-access cocaine self-administration and abstinence. SMAASH-C was developed for use in our clinical practice (Ajibike Salako-Akande) as part of a nutritional approach for managing patients with a substance use disorder. This patented formulation (U.S. Patent Nos. US11,246,892 and US11,890,305; Getwele Natureceuticals, LLC; Halethorpe, MD) contains a combination of vitamins, minerals, omega-3 fatty acids, and tyrosine and other amino acids that are known to be depleted in both humans and animals following chronic cocaine use/exposure [11–16]. Anecdotal reports and open label observational data from patients indicate that oral SMAASH-C (0.1-0.4 g/kg/day; 6-12 months) treatment during abstinence improves markers of general health (e.g., one patient with co-morbid AIDS-related herpes simplex virus infection reporting marked improvement in viral symptoms) and reduces drug-craving, frequency of relapse, and withdrawal symptoms. Results from subsequent preclinical studies were also promising and showed that chronic SMAASH-C treatment (oral, 0.04-0.12 g/kg, 8 weeks) markedly and selectively reduced cocaine- and amphetamine-induced conditioned place preference and markers of drug-induced toxicity in male and female rats [17, 18].

In the present study, effects of SMAASH-C treatment were determined for both cue-induced cocaine-seeking, as a model of drug-craving/vulnerability to relapse, and markers of organ toxicity focusing on serum markers of kidney (urea nitrogen), liver (aspartate transaminase, AST; alanine transaminase, ALT), and pancreas (amylase) functioning. Effects were determined in both males and females and following a two-week treatment regimen at a moderate and moderate-to-high dose (0.4 and 0.8 g/kg/day) and a 6-week regimen at the moderate dose (0.4 g/kg/day). Based on the preliminary clinical and preclinical findings, we predicted that both the 2- and 6-week treatment regimens of SMAASH-C would decrease cocaine-seeking and markers of toxicity.

## Material and methods

### Subjects

Adult male (*N* = 49) and female (*N* = 42) Sprague-Dawley rats (Charles River Laboratories) were housed in individual Med-Associates operant chambers and pre-trained to lever-press for sucrose pellets to expedite subsequent cocaine self-administration training. Rats were also pre-exposed to a jar with ground food and some rats were given sticks to prevent teeth overgrowth. All procedures were approved by the University of Virginia Animal Care and Use Committee and were conducted in accordance with NIH guidelines.

## Procedures

### Surgery and catheter maintenance

An indwelling catheter (Dow Corning, Midland, MI, USA) was implanted into the right jugular vein, and patency was maintained and verified using heparinized saline and methohexital (1.5 mg/kg), respectively [19]. If catheter patency was lost, a left jugular vein catheter was implanted and testing resumed after a 1–2-day recovery period.

### Cocaine self-administration

Rats were trained to self-administer cocaine (1.5 mg/kg/infusion) under a fixed-ratio 1 schedule (FR1; 20), and once acquired (i.e., 2 consecutive days wherein all 20 infusions were obtained), they were given extended-access to cocaine for 10 days using a discrete trial procedure (4, 10-minute trials/hr; up to 96 infusions/day; 7). After the last extended-access session, cocaine was available for one additional session under a FR1 schedule (maximum of 20 infusions) to equate intake between animals prior to abstinence (e.g., all animals obtained all 20 available infusions). Then, a 2- (Experiment 1) or 6-week (Experiment 2) abstinence period began wherein rats remained in their operant chambers with the active lever retracted. Prior to the abstinence period, three males and one female had to be removed from the study because of health or patency issues.

### Experiment 1

**Fig. 1:**
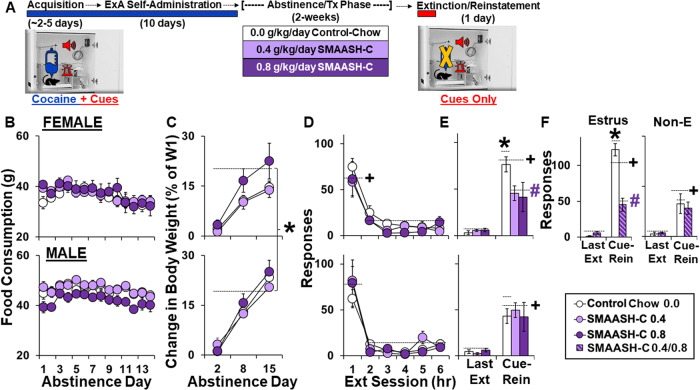
SMAASH-C blunts cue-induced cocaine-seeking in females following a two-week treatment regimen over abstinence. **A** Experimental summary: Male and female rats were trained to self-administer cocaine under a fixed ratio (FR) 1 schedule (acquisition), and once acquired (2 days, 20 infusions), they were given extended-access (ExA, 24-hr/day) to cocaine (4 discrete trials/h, maximum of 96 infusions/day) for 10 days. Rats were given daily treatments (Tx) throughout abstinence with either control-chow (13 males, 10 females) or chow containing a moderate (0.4 g/kg; 11 males, 11 females), or a moderate-to-high dose of SMAASH-C (0.8 g/kg; 8 males, 8 females). Cocaine-seeking was assessed on abstinence day 15 using an extinction/cue-induced reinstatement procedure. **B** Mean daily food consumption over the 15-day abstinence period for females (Upper Panel) and males (Lower Panel) in the control-chow (no SMAASH-C) and the chow plus 0.4 and 0.8 g/kg SMAASH-C groups. **C** Mean change in body weight on abstinence day 2, 8, and 15 (relative to day 1, just prior to SMAASH-C/control treatment) for females (Upper Panel) and males (Lower Panel) in the control-chow and the 0.4 and 0.8 g/kg SMAASH-C groups. **D** Mean responses on the lever formerly associated with cocaine during the six extinction (Ext) sessions for females (Upper Panel) and males (Lower Panel) in the control-chow and the 0.4 and 0.8 g/kg SMAASH-C groups. **E** Mean responses on the lever formerly associated with cocaine during the last extinction (Ext) session versus the reinstatement session (Cue-Rein) for females (Upper Panels) and males (Lower Panels) in the control-chow and the 0.4 and 0.8 g/kg SMAASH-C groups. **F** Mean responses on the lever formerly associated with cocaine during the last extinction (Ext) session versus the reinstatement session (Cue-Rein) for females tested during estrus (Left Panel) versus non-estrus phases (Right Panel) in the control-chow (*n* = 3 estrus, and 5 non-estrus) and the SMAASH-C groups (collapsed across dose; *n* = 5 estrus, and 8 non-estrus). Data are plotted as means ± SEM. *Significant effect of sex or estrous phase (**C**, **E**, **F**). +Significant effect of session (**D**–**F**). #Significant effect of SMAASH-C (**E**, **F**).

Rats in Experiment 1 were randomly assigned to control-chow (14 males, 11 females), or chow containing a moderate SMAASH-C dose (0.4 g/kg; 11 males, 11 females), or a moderate-to-high SMAASH-C dose (0.8 g/kg; 8 males, 8 females; Fig. 1A). These samples sizes were selected based on power analyses and moderate expected effect sizes and the doses were selected because they are analogous to the g/kg doses used clinically (after equating for rat-human differences in body surface area using a conversion factor of 6.2; 21) [20]. SMAASH-C was mixed with ground chow (Teklad LM-485 7912) and water (2:1 ratio), placed in a jar and weighed, and given to the rats daily throughout abstinence and relapse testing. The amount of SMAASH-C added to the food was adjusted throughout abstinence based on level of consumption and body weight to ensure that each rat obtained the appropriate SMAASH-C dose (either 0.4 or 0.8 g/kg/day, ± 0.05 g/kg/day). Control rats received the same ground chow/water mash, but without SMAASH-C.

Drug-seeking was examined on abstinence day 15 using an extinction/cue-induced reinstatement procedure [7–10, 21–23]. Estrous cycle phase on the test day was determined from vaginal cytology samples by an experimenter blinded to the experimental conditions, as previously described [24, 25]. One male and two females in the control-chow group and one female in the 0.4 and 0.8 g/kg SMAASH-C groups were removed from the study because of health issues. One male and one female in the control-chow group and one male and one female in the 0.4 g/kg SMAASH-C group were removed from the study due to patency or technical issues. One female in the 0.8 g/kg SMAASH-C group was a significant Grubb’s outlier on all measures of drug-seeking (extinction, reinstatement, and total seeking) and was excluded from all analyses. This resulted in a final group size of 12 males and 8 females in the control-chow group, 10 males and 9 females in the 0.4 g/kg SMAASH-C group, and 8 males and 6 females in the 0.8 g/kg SMAASH-C group.

**Fig. 2:**
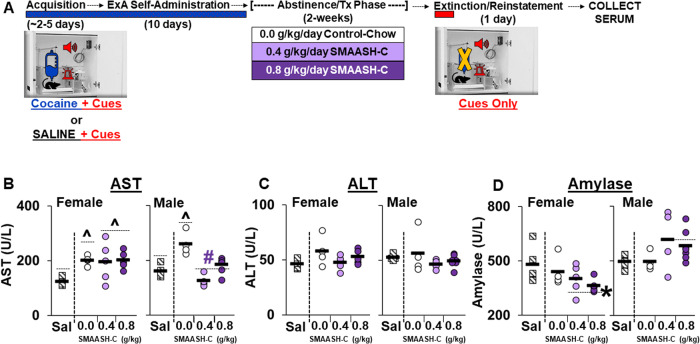
SMAASH-C blunts cocaine/abstinence induced toxicity in males following a two-week treatment regimen. **A** Experimental summary: Serum was collected the morning after extinction/reinstatement testing from a subset of rats in the control-chow (*n* = 13 males, 10 females), 0.4 g/kg SMAASH-C (*n* = 11 males, 11 females), and 0.8 g/kg SMAASH-C (*n* = 8 males, 8 females) treatment (Tx) groups as well as from additional groups of control-chow rats given access to saline (Sal) rather than cocaine during acquisition and the extended-access (ExA) phase (*n* = 5 males and *n* = 6 females). **B** Serum concentration of aspartate transaminase (AST; **B**), alanine transaminase (ALT, **C**), and amylase (**D**) for each of the female (left panels) and male (right panels) rats in the control-chow and 0.4 and 0.8 g/kg SMAASH-C groups. Solid black bars represent group means. ^Significant difference from saline (**B**). #Significant effect of SMAASH-C (**B**). *Significant effect of sex (**D**).

Trunk blood was collected from a subset of rats in the control-chow (4 males, 5 females), 0.4 g/kg SMAASH-C (4 males, 5 females) and 0.8 g/kg SMAASH-C groups (7 males, 5 females) the morning following the extinction/reinstatement test (between 10AM and 12PM) and separated into plasma and serum (19, 25; Fig. 2A). Serum was stored at −80 °C until a small animal chemistry profile examining markers for liver (AST; ALT), pancreases (amylase), and kidney (urea nitrogen) function was completed by Michigan State University’s Veterinary Diagnostics Lab (https://cvm.msu.edu/vdl). An additional cohort of saline rats (5 males, 6 females) were also run under the same conditions as the cocaine group and included as healthy, drug-naive controls. One female in the saline group was a significant Grubb’s outlier for AST and one female in the saline group and one male in the 0.4 g/kg SMAASH-C group were significant Grubb’s outliers for ALT; these rats were excluded from that particular analysis.

### Experiment 2

**Fig. 3:**
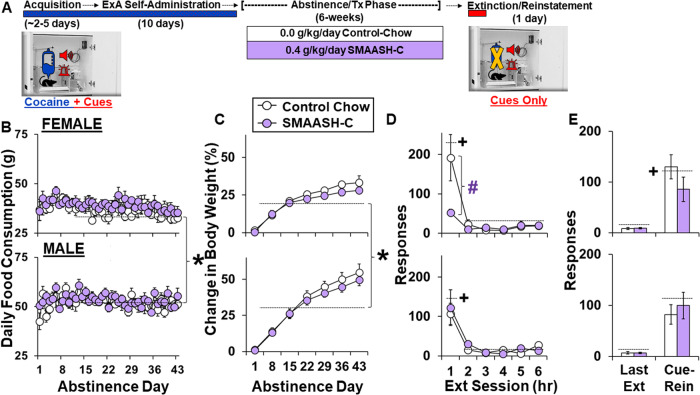
SMAASH-C blunts cocaine-seeking in females during extinction following a six-week treatment regimen over abstinence. **A** Experimental summary: male and female rats were trained to self-administer cocaine under a fixed ratio (FR) 1 schedule (acquisition), and once acquired (2 days, 20 infusions), they were given extended-access (ExA, 24-hr/day) to cocaine (4 discrete trials/h, maximum of 96 infusions/day) for 10 days. Rats were given daily treatments (Tx) throughout abstinence of either control-chow (5 females, 6 males) or chow containing a moderate dose of SMAASH-C (0.4 g/kg; 6 females, 7 males). Cocaine-seeking was assessed on abstinence day 43 using an extinction/cue-induced reinstatement procedure. **B** Mean daily food consumption over the 43-day abstinence period for females (upper panel) and males (Lower Panel) in the control-chow and SMAASH-C groups. **C** Mean change in body weight on abstinence day 2, 8, 15, 22, 29, 36, and 43 (relative to day 1, just prior to SMAASH-C/control treatment) for females (upper panel) and males (Lower Panel) in the control-chow and SMAASH-C groups. **D** Mean responses on the lever formerly associated with cocaine during the six extinction (Ext) sessions for females (upper panel) and males (lower panel) in the control-chow and SMAASH-C groups. **E** Mean responses on the lever formerly associated with cocaine during the last extinction (Ext) session versus the reinstatement session (Cue-Rein) for females (Upper Panels) and males (lower panels) in the control-chow and SMAASH-C groups. Data are plotted as means ± SEM. *Significant effect of sex (**B**, **C**). +Significant effect of session (**D**, **E**). #Significant effect of SMAASH-C (**D**).

**Fig. 4:**
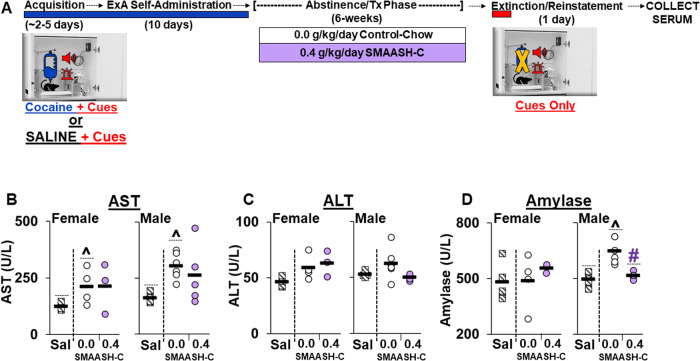
SMAASH-C blunts cocaine/abstinence induced toxicity in males following a six-week treatment regimen. **A** Experimental summary: Serum was collected the morning after extinction/reinstatement testing from a subset of rats in the control-chow (*n* = 5 males, 4 females) and 0.4 g/kg SMAASH-C (*n* = 5 males, 3 females) treatment (Tx) groups as well as from additional groups of control-chow rats given access to saline (Sal) rather than cocaine during acquisition and the extended-access (ExA) phase (*n* = 5 males and *n* = 6 females). **B** Serum concentration of aspartate transaminase (AST; **B**), alanine transaminase (ALT, **C**), and amylase (**D**) for each of the female (Left Panels) and male (Right Panels) rats in the control-chow and 0.4 g/kg SMAASH-C groups. Solid black bars represent group means. ^Significant difference from saline (**B**, **D**). #Significant effect of SMAASH-C (**D**).

The same procedure as detailed in Experiment 1 was in this experiment, except that rats were randomly assigned to receive control-chow (6 males, 5 females) or chow containing 0.4 g/kg SMAASH-C (7 males, 6 females) and relapse testing occurred after a 6-week treatment regimen (day 43; Fig. 3A). Trunk blood was collected the day after the relapse test from a subset of rats in the control-chow (6 males and 4 females) and SMAASH-C groups (5 males and 3 females; Fig. 4A). Serum markers of toxicity were compared to drug-naive controls using the same saline rats as Experiment 1. One male in the 0.4 g/kg SMAASH-C group was a significant Grubb’s outlier for amylase and urea nitrogen and was excluded from these analyses.

## Drug

Cocaine hydrochloride (National Institute of Drug Abuse; Research Triangle Park, NC) was dissolved in sterile saline, stored at 4 °C, and sterile filtered. Infusion duration (2-sec/100 kg) was adjusted three times/week based on body weight. The purity of each component of SMAASH-C (Table 1; also see US 2022/0339204) was verified by AEON Technologies (Frostburg, MD) using a combination of Inductively Coupled Plasma Mass Spectrometry, High Performance Liquid Chromatography with a Diode Array Detector, and Gas Chromatography Mass Spectrometry. SMAASH-C was then compounded by Kydes Pharmaceuticals’ (Halethorpe, MD) and Getwele Natureceuticals (Halethorpe, MD) provided the verified samples tested here.

**Table 1:** Components of the Novel Nutritional Supplement, SMAASH-C.

Test Description	Test Methods	Specification (mg/g)	Test Results (mg/g)
L-Tyrosine	HPLC Martke AA	176	160
Ultra Vitamins	HPLC-Martek WVS & FSV	329	299
Amino Acid Complex	HPLC Martke AA	154	150
Fish Oil (EPA/DHA)	GC FAME	135	123
Vitamin B6	HPLC-Martek WVS & FSV	27	31
Magnesium Oxide	ICP Fullon_04262018	18	20
Calcium Carbonate	ICP Fullon_04262018	56	55

## Data analysis

Repeated measures ANOVA was used to determine group (SMAASH-C versus control-chow) and sex differences in cocaine intake (infusions) over the extended-access period, daily food consumption (with and without SMAASH-C) and changes in body weight over abstinence, and cocaine-seeking over the six extinction sessions, and during reinstatement as compared to the last extinction session run. Changes in body weight were determined relative to abstinence day 1, which was immediately following the last extended-access session and prior to SMAASH-C/control treatment; changes were determined 24-hr after the first treatment (on abstinence day 2), and thereafter, weekly (i.e., on days 8 and 15 for Experiment 1 and on days 8, 15, 22, 29, 36, and 43 for Experiment 2). Univariate analyses were used to determine group (SMAASH-C versus control-chow) and sex differences in serum markers of toxicity (relative and percent difference from saline or control-chow) and abstinence-induced changes in cocaine-seeking (relative to 2-weeks or control-chow), and serum markers of toxicity (relative to saline controls) within males and females. Estrous phase effects (non-estrus versus estrus) were assessed similarly but only for Experiment 1, since Experiment 2 was not powered to address estrous cycle effects (i.e., *n* = 3 estrus and 2 non-estrus, control-chow; *n* = 5 estrus and 1 non-estrus, SMAASH-C). Dose (0.4 and 0.8 g/kg) was considered as a covariant (Experiment 1), but since there were no significant overall or interactive effects of dose on any measure of cocaine-seeking (total and hourly extinction responses, total reinstatement responses, and overall seeking as defined by hour one extinction responses plus reinstatement responses), effects were collapsed across dose (note: Data are presented for each dose separately for clarity). Post-hoc comparisons were made using two-tailed Bonferroni-corrected, two-sample (for pairwise comparisons), or one-sample t-tests (versus no difference, 0, as compared to saline or the 2-week abstinence group). Statistical analyses were performed using SPSS (V26). Alpha was set at 0.05. Data are presented as the mean ± SEM.

## Results

### Experiment 1

#### Cocaine intake, food consumption, & body weights

Cocaine intake over the 10-day extended-access period did not differ between the sexes or within males and females later given control-chow (average infusions/day ± SEM: females, 75 ± 3; males, 71 ± 2) or chow plus 0.4 g/kg SMAASH-C (females, 72 ± 4; males, 72 ± 1), or 0.8 g/kg SMAASH-C (female, 71 ± 2; males, 70 ± 2) indicating that cocaine intake was similar between the sexes and treatment groups prior to abstinence and SMAASH-C/control treatment. Average food consumption during abstinence tended to be higher in males versus females (*p* = 0.06**;** Fig. 1B). However, there were no significant overall or interactive effects of SMAASH-C treatment on food consumption indicating that SMAASH-C supplementation did not impact food consumption. Body weight changes over abstinence also did not differ between treatment groups (non-significant overall and interactive effects of SMAASH-C treatment), but males gained more weight than females (effect of sex, *F*_1,49_ = 4.8, *p* < 0.05), and both males and females gained more weight at later abstinence time-points compared to earlier ones (effect of session, *F*_2,98_ = 242, *p* < 0.001; Fig. 1C). Analysis within each sex similarly revealed an overall effect of session (females, *F*_2,42_ = 57, *p* < 0.001; males, *F*_*2,56*_ = 237, *p* < 0.001), but non-significant overall or interactive effects of SMAASH-C treatment (*p*’s > 0.05) indicating that SMAASH-C did not impact weight gain over abstinence.

#### Relapse vulnerability

Extinction responding was similar between the sexes and treatment groups over the six, 1-hr sessions (Fig. 1D), and all groups responded at higher levels during the first session as compared to later ones (effect of session, *F*_5,24_ = 72.6, *p* < 0.001; session 1 versus 2–6, *p* < 0.001). In contrast, significant effects of SMAASH-C treatment and sex were observed for reinstatement responding (Fig. 1E), with results revealing a significant interaction of session, sex, and SMAASH-C treatment (*F*_1,49_ = 4.6, *p* < 0.05). Within-sex analyses revealed significant effects of session for both females (*F*_1,21_ = 60.0, *p* < 0.001) and males (*F*_1,28_ = 49.8, *p* < 0.001) indicating that responding was reinstated by cocaine-associated cues in both sexes. There was also a significant interaction of session by treatment within females (*F*_1,21_ = 6.0, *p* < 0.05), but not males, with follow-up comparisons revealing a significant sex difference within the control group (F > M) but not the SMAASH-C group, indicating that in females SMAASH-C reduced cocaine-seeking down to the male level.

In females, the efficacy of SMAASH-C at reducing cocaine-seeking varied across the estrous cycle (Fig. 1F). While no phase effects were apparent for extinction responses (data not shown), a significant interaction of estrous cycle phase, session, and treatment was observed for cue-induced reinstatement of cocaine-seeking (*F*_1,48_ = 8.4, *p* < 0.01) which reflects heightened cocaine-seeking in estrus females, and reversal of this effect by SMAASH-C treatment. Post-hoc comparisons confirmed that responding was reinstated by cues in both estrus (*F*_1,8_ = 111, *p* < 0.001) and non-estrus rats (*F*_1,11_ = 28.4, *p* < 0.001), but highest in control-estrus females compared to all other groups (*p*’s < 0.05).

#### Cocaine/abstinence-induced toxicity

In contrast to the behavioral findings, two-weeks of SMAASH-C treatment over abstinence reduced serum makers of organ toxicity in males, but not females. For the liver enzyme AST (Fig. 2B), levels were increased in both females and males (effect of group, *F*_2,33_ = 11.7, *p* < 0.001), and in males, but not females, levels were decreased by SMAASH-C (interaction of sex by treatment, *F*_2,33_ = 5.7, *p* < 0.01). Similarly, within-sex analyses revealed a significant effect of treatment group in both females (*F*_2,16_ = 6.2, *p* = 0.01) and males (*F*_2,17_ = 11.9, *p* < 0.001), but in females, AST levels were higher in both the control-chow and SMAASH-C groups as compared to saline (*p*’s < 0.01), whereas in males, AST levels were higher in the control-chow group compared to both the saline (*p* < 0.001) and SMAASH-C groups (*p* < 0.01)., AST levels in males also did not differ between the SMAASH-C and saline groups indicating this effect of cocaine was normalized by SMAASH-C treatment during abstinence. In contrast, the other liver enzyme assessed, ALT, did not differ by sex or treatment group (Fig. 2C). For the pancreas enzyme amylase, female had lower levels than males (effect of sex, *F*_1,34_ = 10.8, *p* < 0.01), particularly in the SMAASH-C groups (sex by treatment interaction, *F*_2,34_ = 5.9, *p* < 0.01; Fig. 2D). Subsequent within-group comparisons similarly revealed a significant effect of sex within the SMAASH-C group only (*F*_1,19_ = 27.3, *p* < 0.001). There was also a trend for an effect of group in the within-sex analyses, but these effects did not reach statistical significance (females, *p* = 0.06; males, *p* = 0.08). There were no effects of sex or group on levels of urea nitrogen, which is a marker of kidney function (data not shown). Thus, following extended-access cocaine self-administration and 14 days of abstinence, levels of the liver enzyme AST were increased in both males and females and normalized within males, but not females, by SMAASH-C treatment during abstinence. ALT, amylase, and urea nitrogen levels were unchanged following extended-access cocaine self-administration and 14 days of abstinence although a sex difference was apparent for levels of the pancreas enzyme, amylase, with SMAASH-C treated females having lower levels than their male counterparts.

### Experiment 2

#### Cocaine intake, food consumption, & body weights

As with experiment 1, no sex or group differences were observed for cocaine intake over the 10 extended-access sessions (average infusions/day±SEM: females, 76 ± 3; males, 70 ± 4) or 0.4 g/kg SMAASH-C (females, 75 ± 1; males, 75 ± 1) indicating that the groups were similar with regard to cocaine exposure prior to abstinence and SMAASH-C/control treatment. While females consumed less food over the 6-week period of abstinence compared males (effect of sex, *F*_1,20_ = 121.6, *p* < 0.001), there were no significant overall or interactive effects of SMAASH-C treatment in the overall or within-sex analyses (Fig. 3B). Females also gained less weight over the 6-week abstinence period than males (effect of sex, *F*_1,20_ = 17.3, *p* < 0.001; session by sex, *F*_6,120_ = 28.1, *p* < 0.001), but there were no significant overall or interactive effects of SMAASH-C treatment on body weight in the overall or within-sex analyses (Fig. 3C).

#### Relapse vulnerability

In contrast to Experiment 1, significant sex/group differences were observed for extinction responding in this experiment following 6-weeks of control versus SMAASH-C treatment during abstinence (Fig. 3D) with results showing that SMAASH-C decreased extinction responding in females, but not males, particularly during the first session, when levels of responding were highest (effect of session (*F*_5,100_ = 27.7, *p* < 0.001; sex by session by treatment, *F*_5,100_ = 4.0, *p* < 0.05). Subsequent analysis within females revealed both a session (*F*_5,45_ = 14.5, *p* < 0.001) and session by treatment group interaction (*F*_5,45_ = 6.2, *p* < 0.001) which appears to be driven by lower responding in SMAASH-C versus control-chow females in session one (*p* < 0.05). Analysis within groups also confirmed a session effect and higher responding in session 1 within the control-chow group (*F*_5,45_ = 6.2, *p* < 0.001; *p’s* < 0.05, respectively), but not the SMAASH-C group. In contrast, this same analysis in males confirmed that responding was highest in the first session (effect of session, *F*_5,55_ = 13.6, *p* < 0.001; session 1 versus 2-6, *p*’s < 0.001), but not impacted by SMAASH-C treatment (no overall or interactive effects of treatment group). No significant effects of sex or treatment were observed for reinstatement responding following 6-weeks of abstinence and control/SMAASH-C treatment (Fig. 3E), and all groups showed a similar reinstatement effect in response to the cocaine-associated cues (effect of session*, F*_1,20_ = 59.2, *p* < 0.001). Thus, SMAASH-C decreased cocaine-seeking in females during the first extinction session when it was highest, but did not impact reinstatement responding in either females or males.

#### Cocaine/abstinence-induced toxicity

As in Experiment 1, six-weeks of SMAASH-C treatment over abstinence improved markers of cocaine-induced toxicity in males, but not females. However, in contrast to Experiment 1, in this experiment, effects were most apparent for amylase, the marker of pancreatic toxicity. Specifically, AST levels were similarly elevated in females and males in the SMAASH-C and control-chow groups (effect of group, *F*_2,22_ = 6.2, *p* < 0.01; SMAASH-C versus control-chow, *p* > 0.05; Fig. 4B). The increase in AST was more variable in the SMAASH-C group, however, with post-hoc comparisons to saline revealing a significant difference for the control-chow group (*p* < 0.05), but not only trend for the SMAASH-C (*p* = 0.072). Females also tended to have lower overall levels of AST than males (*F*_1,22_ = 3.9, *p* = 0.06). For ALT, levels tended to be higher in the control-chow and SMAASH-C groups compared to the saline group (Fig. 4C), but the overall effect of group did not reach statistical significance (*p* = 0.053). For amylase, there was an interaction of sex and treatment group (Fig. 4D; *F*_2,21_ = 3.8, *p* < 0.05) and subsequent within-sex analyses revealed a significant effect of treatment group in males (*F*_2,12_ = 15.5, *p* < 0.001), but not females, which reflects higher amylase levels in the control-chow group as compared to both the saline (*p* < 0.05) and SMAASH-C (*p* < 0.05) groups. Notably, there was no difference in amylase levels between the saline and SMAASH-C groups indicating that in males SMAASH-C treatment normalized cocaine-induced increases in amylase. There were no effects of sex or group on levels of urea nitrogen, the marker of kidney function (data not shown). Thus, following extended-access cocaine self-administration and 6 weeks of abstinence, levels of the liver enzymes AST were increased in both males and females. Levels of the pancreatic enzyme amylase also increased in males but were normalized by SMAASH-C treatment during abstinence.

## Summary

**Fig. 5:**
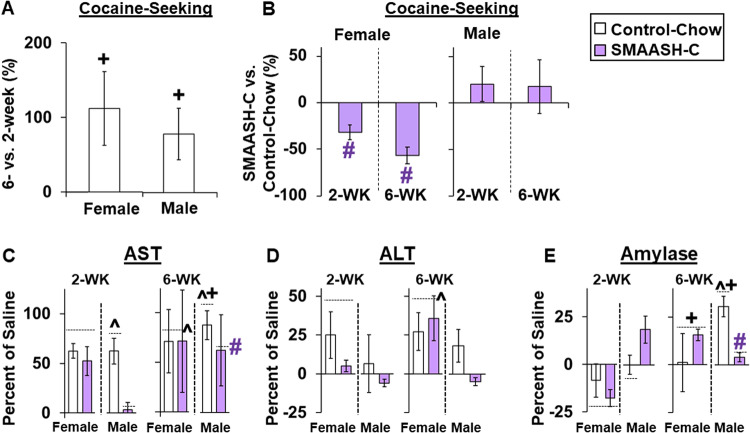
A summary of the behavioral and toxicity findings across both experiments. Mean (±SEM) percent difference from the 2-week. Mean (±SEM) percent difference from the 2-week (**A**) and control-chow (**B**) group for cocaine-seeking (responses during hour one of extinction plus reinstatement), and from the saline control group for serum levels of aspartate transaminase (AST; **C**), alanine transaminase (ALT, **D**), and amylase (**E**). +Significant difference from 2-weeks (**A**, **C**, **E**). #Significant effect of SMAASH-C (**B**, **C**, **E**). ^Significant difference from saline (or 0; **C**–**E**).

A summary of the behavioral and toxicity findings across both experiments is shown in Fig. 5 with data presented as percent difference from the 2-week (A), control-chow (B), and saline control groups (C–E). Our behavioral findings show that cocaine-seeking (i.e., total responses during reinstatement plus the first extinction session) was higher in females and males tested following six-versus two-weeks of abstinence (or 0, *p*’s < 0.05; Fig. 5A), and that in females, but not males, both the 2- and the 6-week SMAASH-C treatment regimens decreased cocaine-seeking relative to control-chow females (or 0, *p*’s < 0.01; Fig. 5B).

For AST, levels increased in males from 2- to 6-weeks (effect of abstinence group, *F*1,22 = 6.0, *p* < 0.05), and were similarly decreased following 2 and 6 weeks of SMAASH-C treatment (overall effect of treatment, *F*1,22 = 5.4, *p* < 0.05; Fig. 5C). AST levels were also significantly higher than saline control values (versus 0) within the control-chow (*p*’s<0.05), but not SMAASH-C groups. For females, AST levels were persistently elevated across groups (versus saline, or 0, *p* < 0.05), but did not further increase over abstinence and were not impacted by SMAASH-C treatment. Two and 6-weeks of SMAASH-C treatment also tended to decrease ALT levels in males, but this effect did not reach statistical significance (effect of group, *p* = 0.051; Fig. 5D). However, ALT levels were not significantly different from saline control values for males in either the control-chow or SMAASH-C groups and did not change significantly over abstinence. In contrast, ALT levels in females were increased from saline control values, but levels did not differ between abstinence or treatment groups (*p* < 0.01). For amylase, levels in females increased from 2 to 6 weeks (abstinence group effect, *F*_1,17_ = 6.1, *p* < 0.05), but they were not significantly impacted by SMAASH-C treatment, and were not higher than saline control values even at the 6-week time-point (Fig. 5E). Levels of amylase also increased over abstinence in males, but in contrast to effects in females, levels at the 6-week time-point were significantly higher than saline control values (in control-chow rats only; versus saline, or 0, *p* < 0.05), and SMAASH-C treatment during abstinence prevented this increase (abstinence by treatment group interaction, *F*_1,21_ = 8.2, *p* < 0.01).

Thus, cocaine-seeking incubated in both males and females over abstinence, and in females, SMAASH-C effectively reduced cocaine-seeking at both time-points. Both males and females showed elevated markers of organ toxicity following abstinence and these effects persisted and/or intensified over abstinence. In males, SMAASH-C effectively reduced markers of cocaine-induced toxicity, with normalization observed for AST following SMAASH-C treatment, particularly the 2-week treatment, and for amylase following the 6-week treatment.

## Discussion

The goal of the present study was to determine the efficacy of oral treatment during abstinence with a novel nutritional supplement, SMAASH-C, on markers of relapse vulnerability and cocaine/abstinence-induced toxicity. We examined effects in both males and females and found that SMAASH-C treatment during abstinence reduced cocaine-seeking in females and markers of cocaine-induced toxicity in males. In females, SMAASH-C treatment over a two-week abstinence period reduced drug-seeking in response to cocaine-associated cues, particularly when females were tested during estrus, when levels of responding are heightened; whereas, treatment over a 6-week abstinence period reduced drug-seeking during extinction, particularly during the first extinction session when responding was highest. Despite its lack of efficacy to reduce drug-seeking in males, SMAASH-C treatment normalized cocaine-induced increases in the liver enzyme AST, particularly at the 2-week time-points, and prevented the abstinence-induced increase in the pancreatic enzyme amylase in males. Thus, the beneficial effects of SMAASH-C treatment during abstinence appears to be sex-specific.

To our surprise, following extended-access cocaine self-administration, SMAASH-C treatment over abstinence reduced cocaine-seeking in females, but not males. The sex-specific efficacy of SMAASH-C appears to be robust considering that these effects were observed following both two- and six-weeks of treatment. SMAASH-C was also most effective at attenuating cocaine-seeking in females tested during estrus (Experiment 1), when relapse vulnerability is heightened [24–27]. Effects were also most apparent following 6-weeks of abstinence during the first extinction session, also when levels of cocaine-seeking were highest [28, 29], suggesting that SMAASH-C treatment may blunt craving during periods of heightened vulnerability. Males also showed heightened vulnerability, particularly following 6-weeks of abstinence, so it is not clear why a similar effect was also not observed in males. It is worth noting, however, that while there was no overall sex difference at the 6-week time-point, levels of cocaine-seeking tended to be higher in control-chow females versus males (321 versus 189 responses, *p* = 0.063); a sex difference was also apparent following 2 weeks of abstinence. Thus, it is possible that SMAASH-C would effectively decrease cocaine-seeking in males as well if vulnerability was further enhanced (e.g., by stress or by testing at later time-points during abstinence). This idea is also consistent with our observational data in humans, which have included both men and women; however, future studies are needed to address this possibility.

**Fig. 6:**
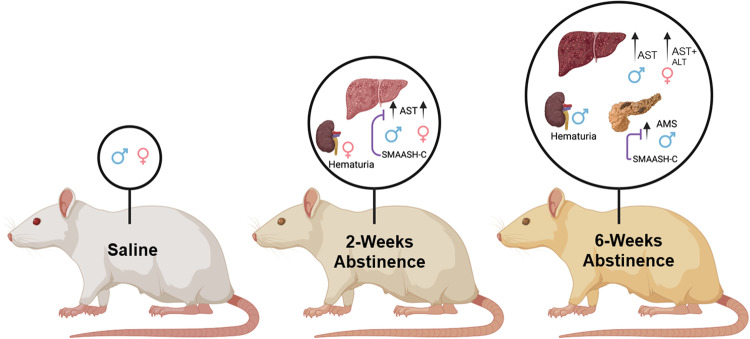
A graphical summary of toxicity changes over abstinence. In drug-naive saline controls no organ toxicity would be expected. In contrast, aspartate transaminase (AST) serum levels, which is a marker of liver damage, was elevated in both males (*n* = 4) and females (*n* = 4) 2-weeks following extended-access cocaine self-administration. SMAASH-C blocked the effect in males (*n* = 11). Also, in two-week abstinence cohort, hematuria was observed in one female. In the 6-week group, AST was elevated in males (*n* = 10) and females (*n* = 7); alanine transaminase (ALT), another marker of liver damage, serum levels were elevated in females (*n* = 7) across the 2- and 6-weeks groups. Amylase serum levels (a marker for pancreatic dysfunction) were increased in males (*n* = 6), and SMAASH-C blocked the effect (*n* = 4). Also found in 6-week group, one male (*n* = 1) with hematuria.

Notably, over the 2- and 6-week cocaine abstinence periods we observed signs of extreme toxicity with one male and two females in the control-chow group and two females in the SMAASH-C group spontaneously developing unexplainable, life-threating illnesses that resulted in their removal from the study (or death). We also observed blood in the urine of a female and male in the control-chow group that had been withdrawn from cocaine for 6 and 37 days, respectively. The follow-up urinalysis for the male indicated kidney failure. These observations lead us to assess serum markers for organ health. Surprisingly, the results revealed no difference in the marker for kidney toxicity (urea nitrogen); however, urea nitrogen is not the preferred marker for renal function and, unfortunately, the small animal chemistry panel was not sensitive enough to detect individual differences in creatinine levels. Notably, the panel did reveal marked differences in two makers of liver toxicity (AST and ALT) in females and one in males (AST) following two- and six-weeks of cocaine abstinence as well as signs of pancreatic toxicity in males following six-weeks of cocaine abstinence (Fig. 6). These results align with findings in humans which have shown that cocaine use can induce extreme hepatoxicity accompanied with high level of aminotransferases (AST and ALT), which is often accompanied by other organ involvement and can be fatal [30].

Surprisingly, the beneficial effects of SMAASH-C treatment on cocaine-induced toxicity were only apparent in males with SMAASH-C treatment improving a marker of liver toxicity (AST), particularly at the 2-week time-point, and six weeks of SMAASH-C treatment improving a marker of pancreatic toxicity (amylase). These findings are also consistent with the sex difference we noted from daily health observations where severe health complications led to the removal of both males and females in the control-chow group; whereas, among SMAASH-C treated rats, only females developed severe health complications indicating that SMAASH-C treatment may improve the overall health of males, but not females, during cocaine abstinence. A previous study reported that SMAASH-C offers protective effects against chronic cocaine exposure by increasing plasma clearance of cocaine and its toxic metabolites through increasing the activity of the liver enzyme CYP450 [18]. One caveat is that while both males and females were included in this previous study, sex was not considered so it is still possible that effects on liver activity were driven by males. Future research is needed to address this question and to determine the mechanisms underlying these sex-specific health benefits of SMAASH-C. One possibility worth consideration is that since the course for development of drug-related health consequences is accelerated in females [31, 32], it is possible that cocaine-induced liver damage may have been more severe/occurred earlier and thus less responsive to treatment over abstinence. This idea is also supported by our current findings showing that by 6 weeks, SMAASH-C is no longer effective at reducing abstinence-induced increases in AST in males along with our observation of blood in the urine at an earlier time-point during abstinence in the control female versus male.

In summary, the present findings validate anecdotal reports and open label observational data in humans indicating that SMAASH-C reduces relapse vulnerability and improves the health of patients with CUD. SMAASH-C is in an oral, non-prescription treatment, and as such, it would be relatively easy to administer at home and have few barriers to access for patients with CUD. Future studies are needed to determine whether SMAASH-C also reduces vulnerability to other aspect of the disease process, such as motivation for cocaine and compulsive use. It would also be helpful to understand the mechanisms underlying the sex-specific benefits; however, there are many treatments used in practice today that we do not understand the mechanism of action. Therefore, this lack of understanding should not delay further exploration of the beneficial application of SMAASH-C in patients with CUD and potentially other substance use disorders.

## Data Availability

The raw data supporting the conclusions of this manuscript will be made available by the authors, without undue reservation, to any qualified researcher.
